# Glomerulonephritis during Mycobacterium tuberculosis infection: scoping review

**DOI:** 10.1186/s12882-024-03716-6

**Published:** 2024-08-31

**Authors:** Adam Forster, Natasha Sabur, Ali Iqbal, Stephen Vaughan, Benjamin Thomson

**Affiliations:** 1 Division of Nephrology, Department of Medicine, Western University, London, ON Canada; 2 Division of Pulmonary Medicine, St. Michael’s Hospital and West Park Healthcare Centre, University of Toronto, Toronto, ON Canada; 3 Division of Nephrology, Department of Medicine, McMaster University, Hamilton, ON Canada; 4 Division of Infectious Diseases, University of Calgary, Calgary, AB Canada; 5 Bloomberg School of Public Health, Johns Hopkins University, Baltimore Maryland, USA

**Keywords:** Glomerulopathy, Nephrotic syndrome, Glomerulonephritis, Nephritic syndrome, Mycobacterium tuberculosis, Multidrug resistant tuberculosis

## Abstract

**Introduction:**

People with Tuberculosis (TB) infection may present with glomerulonephritis (GN). The range of presentations, renal pathologies, and clinical outcomes are uncertain. Whether clinical features that establish if GN etiology is medication or TB related, and possible benefits of immunosuppression remain uncertain.

**Methods:**

A scoping review was completed, searching MEDLINE, EMBASE, Cochrane Central Register of Controlled Trials, Web of Science and Conference Abstracts from Inception to December, 2023. The study population included patients with TB infection who developed GN and underwent renal biopsy. All data regarding presentation, patient characteristics, renal pathology, management of TB and GN, and outcomes were summarized.

**Results:**

There were 62 studies identified, with 130 patients. These cases included a spectrum of presentations including acute kidney injury, nephrotic syndrome and hypertension, and a range of 10 different renal pathology diagnoses. Cases that included immunosuppression and outcomes ranged from complete remission to long-term dialysis dependence. The presence of granulomas (4/4, 100%), anti-glomerular basement membrane disease (3/3, 100%), amyloidosis (75/76, 98.7%), and focal segmental glomerulosclerosis (2/2, 100%) were specific for GN being TB-infection related. On the other hand, minimal change disease was specific for anti-TB therapy related (7/7, 100%). While patients with more aggressive forms of GN commonly were prescribed immunosuppression, this study was unable to confirm efficacy. Only rifampin or isoniazid were implicated in drug-associated GN.

**Discussion:**

This study provides a clear rationale for renal biopsy in patients with TB and GN, and outlines predictors for the GN etiology. Thus, this study establishes key criteria to optimize diagnosis and management of patients with TB and GN.

**Supplementary Information:**

The online version contains supplementary material available at 10.1186/s12882-024-03716-6.

## Introduction

Annually, *Mycobacterium tuberculosis* (TB) infections infect over 10 million people [1], and represent the 13th leading cause of death worldwide [2]. Urogenital TB commonly involves the urinary collecting system, but renal parenchymal involvement has been reported, including interstitial nephritis and glomerulonephritis (GN) [3]. Optimal management of GN during TB infection remains unclear. Identifying whether GN is primary or secondary, and the role of immunosuppression, is uncertain [4, 5]. It is not always possible to identify whether TB infection or anti-tuberculous therapy (ATT) is the precipitant of GN [6–10]. Tuberculosis often coexists with other comorbidities that can precipitate GN, such as human immunodeficiency virus (HIV) [11] or sickle cell disease; [12] and how to isolate the relative contributions of each comorbid condition is unclear.

There is a wide range of glomerular presentations described in TB infection, including IgA nephropathy [13], membranous nephropathy [14], amyloidosis [15], minimal change [16] and membranoproliferative disease [8]. However, there remains a lack of consensus on how to manage these cases. Improved management in cases of glomerular disease in patients with TB may enhance renal and TB-related outcomes.

This scoping review had four objectives. First, we describe the presentation of glomerulonephritis in patients with TB infection. Secondly, we describe the range of renal pathology diagnoses in patients with TB infection and GN. Thirdly, we evaluate which renal pathology features are predictive of whether the GN is from TB versus medication-related. Finally, we evaluate the impact of immunosuppression on clinical outcomes.

## Methods

Our protocol was developed using the recommended methodology for scoping reviews from the Joanna Briggs Institute [17]. 

### Search strategy and study selection criteria

A scoping review was performed using Cochrane, Embase, Medline and Web of Science databases, with no date or language restriction. Search terms included “Glomerulonephritis,” “Glomerulopathy,” “Glomerular disease,” “Nephrotic syndrome,” “Nephritic syndrome,” “Tuberculosis,” “Mycobacterium tuberculosis,” and “Mycobacteria.” Searches were completed using [“Glomerulonephritis” OR “Glomerulopathy” OR “Glomerular disease” OR “Nephrotic syndrome” OR “Nephritic syndrome”] AND [“Mycobacterium tuberculosis” OR “Tuberculosis” OR “Mycobacteria.”].

Studies were included if they described a case of glomerular disease in a patient with TB infection. Renal biopsy matched to the clinical description was mandatory. Adult patients (age 18 or over) were included. All study types were included. References from review articles were screened to assure all cases were identified. Pediatric cases were excluded. Cases in which a renal biopsy was either not performed, or was not matched to the clinical conditions (quantitation of proteinuria, presence of edema or hypertension or acute kidney injury (AKI), etc.) were excluded.

Two authors (BT, AF) screened all titles and abstracts, and completed full text review, using Covidence systematic review software (Veritas Health Innovation, Melbourne, Australia). Conflicts were resolved by discussion and consensus between both authors (BT, AF).

### Data extraction

## Results

**Fig. 1:**
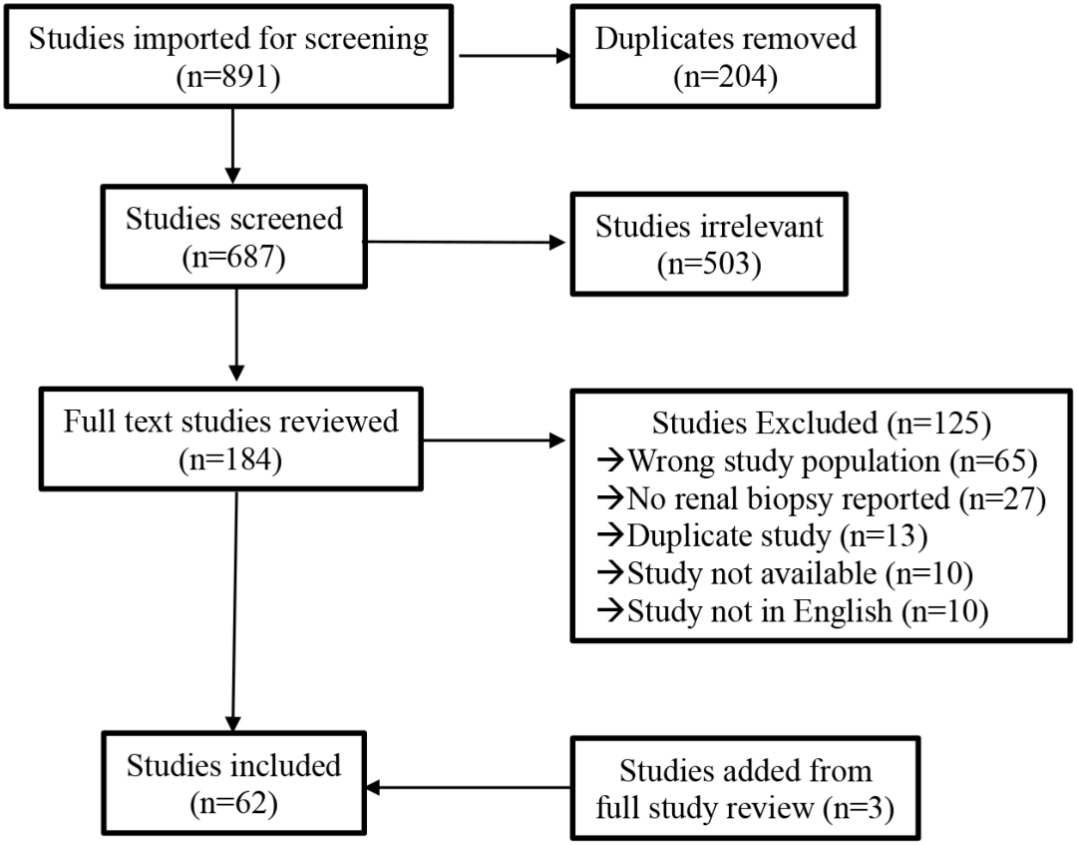
PRISMA chart for study inclusion

Search of databases yielded 891 references (Fig. 1). After duplicates were removed (*n* = 204), study titles and abstracts were screened, leaving full text studies for review (*n* = 184). Additional studies were excluded (*n* = 125), and 3 studies were added as a result of review of references from review papers. This left 62 studies for final inclusion, which included 130 patients.

### Study characteristics

Most included studies were case reports (55/62, 90.3%) but case series represented a higher proportion (74/130, 56.9%) of patients (Table 1). Most included patients were reported between 2000 and 2019 (103/130, 78.5%), with few studies published before 1990 (10/62, 16.1%). Although most studies were published in high income countries (34/62, 54.8%), the majority of patient cases were from low and middle income countries (96/130, 73.8%), with India as the country with the most studies (14/62, 22.6%) and patients (81/130, 62.3%).

**Table 1:** Study characteristics

Characteristic	Studies % (*n*)	Patients % (*n*)
** Total**	**100.0 (62)**	**100.0 (130)**
**Study type**		
Case report	90.3 (56)	43.1 (56)
Case series	9.7 (6)	56.9 (74)
**Year**		
Before 1990	16.1 (10)	8.5 (11)
1990–1999	12.9 (8)	6.2 (8)
2000–2009	17.7 (11)	40.8 (53)
2010–2019	38.7 (24)	37.7 (49)
2020-present	14.5 (9)	6.9 (9)
**Country**		
** High Income Country**	**54.8 (34)**	**26.2 (34)**
Australia	1.6 (1)	0.8 (1)
Canada	1.6 (1)	0.8 (1)
France	3.2 (2)	1.5 (2)
Iran	1.6 (1)	0.8 (1)
Ireland	3.2 (2)	1.5 (2)
Italy	1.6 (1)	0.8 (1)
Japan	12.9 (8)	6.2 (8)
South Korea	4.8 (3)	2.3 (3)
Spain	4.8 (3)	2.3 (3)
Taiwan	4.8 (3)	2.3 (3)
United Kingdom	6.5 (4)	3.1 (4)
United States	8.1 (5)	3.8 (5)
** Low or Middle Income Country**	**45.2 (28)**	**73.8 (96)**
Brazil	3.2 (2)	1.5 (2)
China	1.6 (1)	0.8 (1)
India	22.6 (14)	62.3 (81)
Not stated	1.6 (1)	0.8 (1)
Pakistan	3.2 (2)	1.5 (2)
Papua New Guinea	3.2 (2)	2.3 (3)
South Africa	4.8 (3)	2.3 (3)
Turkey	4.8 (3)	2.3 (3)

### Presentation of glomerular disease in tuberculosis

#### Patient characteristics

Reporting was incomplete for patient age (77.9%) and sex (97.7%). Mean age was 42.3 years and females were a minority of reported cases (27.3%) (Table 2).

**Table 2:** Patient characteristics and clinical presentation

Patient characteristics	Mean (range)
** Age (years)**	42.3 (20–82)
	**% Female (n)**
** Sex**	27.3 (35)
**Clinical Presentation**	**Patients % (n)**
** TB symptoms to GN symptoms (weeks)**	
Reported	33.1 (43)
GN presented before TB symptoms	9.3 (4)
GN presented simultaneous to TB symptoms	20.9 (9)
1–4 weeks	20.9 (9)
5–12 weeks	25.6 (11)
13–24 weeks	16.3 (7)
> 24 weeks	7.0 (3)
** From TB symptoms to GN diagnosis (weeks)**	
Reported	40.8 (53)
GN presented before TB diagnosis	11.1 (6)
GN presented simultaneous to TB diagnosis	43.4 (23)
1–4 weeks	17.0 (9)
5–12 week	11.3 (6)
13–24 weeks	11.3 (6)
> 24 weeks	5.7 (3)
** Tuberculosis Organ Involvement**	
Reported	43.5 (57)
Anus	1.8 (1)
Arthritis	1.8 (1)
Cecum	1.8 (1)
Liver	3.5 (2)
Lung	78.9 (45)
Lymph nodes	8.8 (5)
Peritoneum	1.8 (1)
Urogenital	7.0 (4)
Skin	3.5 (2)
Spine	3.5 (2)
** Clinical signs at presentation**	
24 h urine protein (g/day)	
< 3 g/day	33.1 (43)
3–10 g/day	26.9 (35)
> 10 g/day	16.9 (22)
> 3 g/day (but not otherwise specified)	16.2 (21)
Not stated, but did not have nephrotic syndrome	6.9 (9)
	**% (n/reported)**
Nephrotic syndrome	56.7 (59/104)
Hypertension	42.1 (32/76)
Acute Kidney injury	69.8 (87/126)

### Clinical presentation of glomerulonephritis in patients with tuberculosis

GN presented after TB symptoms in most reported cases (90.7%), and rarely beyond 24 weeks from onset of TB symptoms (7.0%) (Table 2). GN presented after TB diagnosis in most (88.9%) cases, and rarely after 24 weeks from TB diagnosis (5.7%).

**Fig. 2:**
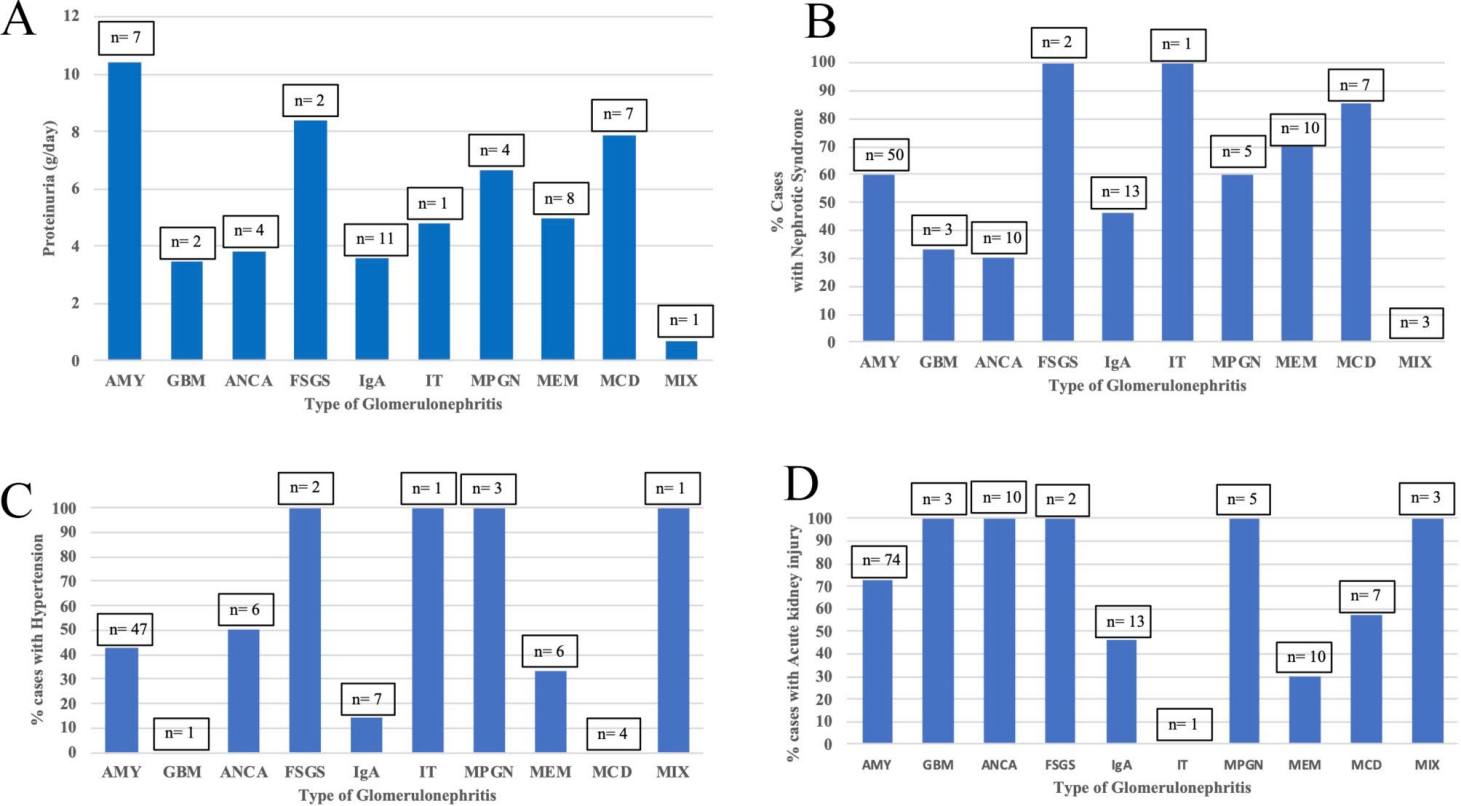
**A** 24 h protein excretion versus Type of Glomerulonephritis **B** Percent cases with Nephrotic syndrome versus Type of Glomerulonephritis **C** Percent cases with Hypertension versus Type of Glomerulonephritis **D** Percent cases with Acute kidney injury versus Type of Glomerulonephritis LEGEND: AMY = amyloidosis; GBM = Anti-GBM antibody disease; ANCA = pauci-immune GN, FSGS = focal segmental glomerulosclerosis; IgA = IgA nephropathy; IT = immunotactoid GN; MPGN = membranoproliferative glomerulonephritis; MEM = membranous nephropathy; MCD = minimal change disease; MIX = mixed pathology

“Reporting of proteinuria quantitation was incomplete, but mean 24 hour proteinuria was highest for amyloidosis (10.4 g/day, *n* = 7 reported), FSGS (8.4 g/day, *n* = 2 reported) and minimal change disease (7.9 g/day, *n* = 7 reported) (Fig. 2A). Nephrotic syndrome was common (60.0%, 78/130 total reported), and was most common in FSGS (100%, *n* = 2 reported), Immunotactoid GN (100%, *n* = 1 reported) and minimal change disease (85.7%, *n* = 7 reported) (Fig. 2B).

Hypertension was common (42.1%), and was found in all cases of FSGS (100%, *n* = 2 reported), Immunotactoid GN (100%, *n* = 1 reported), MPGN (100%, *n* = 3 reported), and mixed pathology (100%, *n* = 1 reported) (Fig. 2C).

Acute kidney injury was common (69.8%), and was identified in all cases of Ant-GBM disease (100%, *n* = 3 reported), pauci-immune GN (100%, *n* = 10 reported), FSGS (100%, *n* = 2 reported), MPGN (100%, *n* = 5 reported) and mixed pathology (100%, *n* = 3 reported) (Fig. 2D).”

The initial site of organ involvement of TB was incompletely reported (43.5%, 57/130). The most common site of reported TB organ involvement was lung (78.9%), with lymphadenopathy (8.8%) and renal-limited (7.0%) next most common. Only 1 case explicitly described disseminated TB, but there was insufficient description to rule this out in other cases.

### Renal biopsy: general features

Whether crescents were present on renal biopsy could be determined in 59 of 62 studies, and each of these 59 studies included 1 case each. Crescents were commonly identified (20/59, 33.9%) (Appendix 1). In addition to being found in all cases of crescentic (pauci-immune) GN (10/10), crescents were also identified in Anti-GBM disease (3/3), IgA nephropathy (3/13, with 2/3 Henoch Schoenlein purpura (HSP) cases), Membranoproliferative glomerulonephritis (MPGN) (3/5), and mixed pathology with features of IgA/MPGN/Membranous nephropathy (1/3). Patients with crescents on renal biopsy had GN attributed to medications in 4 cases (4/20, 20.0%); this included crescentic pauci-immune GN (3/10) and MPGN (1/5) (Table 3, Appendix 1).

**Table 3:** Renal pathology and attribution to medication

Pathology	Cases (*n*)	Attributed to medication (%)	Medication (*n* cases)	AIN (%)	Granulomas (%)	Crescents (% )
Amyloidosis	76	1.3	Uncertain	0.0	0.0	0.0
Anti-Glomerular Basement Membrane	3	0.0	NR	0.0	0.0	100.0
Crescentic GN	10	30.0	RIF (3)	50.0	0.0	100.0
Focal Segmental glomerulosclerosis	2	0.0	NR	0.0	0.0	0.0
IgA nephropathy	13	0.0	NR	7.7	7.7	23.1*
Immunotactoid	1	0.0	NR	0.0	0.0	0.0
Membranoproliferative	5	20.0	RIF (1)	0.0	0.0	60.0
Membranous	10	20.0	RIF (1), RIF or ISN (1)	60.0	30.0	0.0
Minimal change disease	7	100.0	RIF (4), ISN (1), RIF or ISN (2)	42.9	0.0	0.0
Mixed (Features of Membranous, IgA and MPGN)	3	33.3	RIF (1)	0.0	0.0	33.3

ISN = isoniazid; NR = not relevant; NS = not stated; RIF = rifampin

* 2 of these cases were in patients with Henoch-Schonlein Purpura

Granulomas were rarely reported (Appendix 1) (4/59, 6.8%), in IgA nephropathy (1/13), and membranous nephropathy (3/13). Patients with granulomas on renal biopsy had GN attributed to TB in all cases (4/4).

AIN was common (Appendix 1) (15/59, 25.4%), in crescentic (pauci-immune) GN (5/10), IgA nephropathy (1/13 cases), membranous nephropathy (6/10) and minimal change disease (3/7). Patient with AIN on renal biopsy had GN attributed to medication exposure in 6 cases (6/15, 40.0%); this included minimal change disease (3/7), Crescentic pauci-immune GN (2/10), and membranous nephropathy (1/10).

### Cases with urogenital tuberculosis as primary site

There were four cases in which urogenital system was considered the primary site of infection of tuberculosis. The cases included membranous nephropathy (3/4) and IgA nephropathy (1/4).

The three cases of membranous nephropathy presented with nephrotic syndrome (3/3), and also had AIN (3/3) and granulomas (2/3) on renal biopsy. The case of IgA nephropathy presented with AKI, hematuria and subnephrotic-range albuminuria. There was no AIN, crescents or granulomas on renal biopsy.

All four cases were attributed to tuberculosis infection, with GN improving with ATT.

### Attribution of glomerulonephritis to medication

**Fig. 3:**
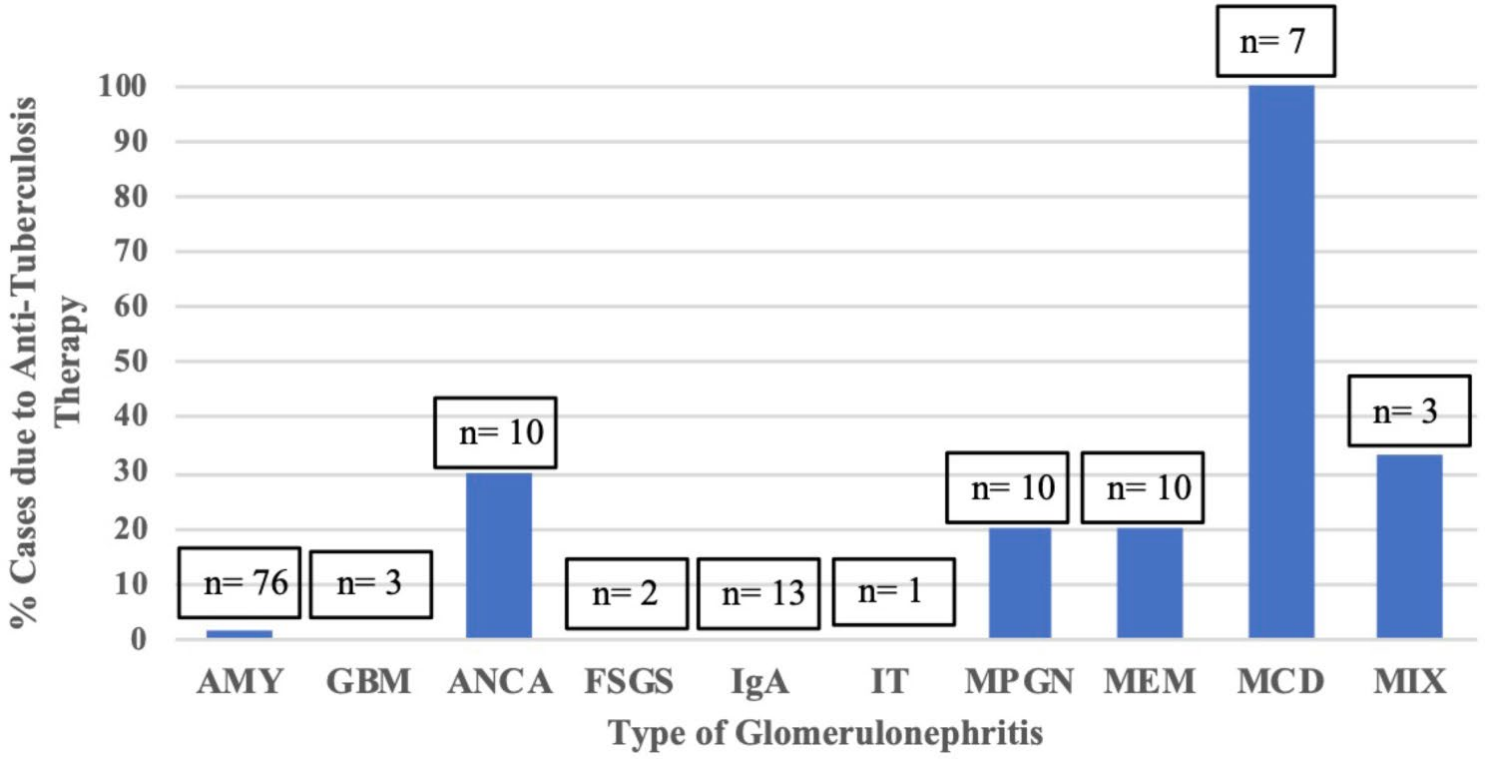
Percent cases attributed to Anti-Tuberculosis Therapy versus Type of Glomerulonephritis LEGEND: AMY = amyloidosis; GBM = Anti-GBM antibody disease; ANCA = pauci-immune GN, FSGS = focal segmental glomerulosclerosis; IgA = IgA nephropathy; IT = immunotactoid GN; MPGN = membranoproliferative glomerulonephritis; MEM = membranous nephropathy; MCD = minimal change disease; MIX = mixed pathology

Attribution of GN to Anti-Tuberculosis medication was most common in minimal change disease (100%, 7/7 reported), but also was found in crescentic pauci-immune GN (30%, 3/10 reported), MPGN (20%, 2/10 reported) and membranous nephropathy (20%, 2/10 reported) (Fig. 3).

Reporting of medications used in ATT was complete (Appendix 2) in 13/15 (86.7%) and partially complete in 1 (6.7%) cases. The most commonly prescribed ATT medications were rifampin (13/13) and isoniazid (13/13), followed by pyrazinamide (11/13), ethambutol (8/13), levofloxacin (1/13), streptomycin (1/13) and clarithromycin (1/13).

When GN was medication-associated (Appendix 2), rifampin (10/15), isoniazid (1/15), either isoniazid or rifampin (3/15) or unknown (1/15) was implicated. No drug other than isoniazid or rifampin was implicated in medication-associated GN in patients with TB.

### Use of immunosuppression

Combined use of corticosteroids with cyclophosphamide and plasmapheresis was reserved for cases (Appendix 2) of Anti-GBM (3/3) and pauci-immune crescentic GN (1/10). Corticosteroids alone were used in pauci-immune crescentic GN (4/10), Focal segmental glomerulosclerosis (FSGS) (2/2), IgA nephropathy (3/13), Membranous nephropathy (3/10), MPGN (1/5) and minimal change disease (3/7).

When AIN was present on renal biopsy, immunosuppression was commonly prescribed (Appendix 2) (7/15, 46.7%). Improvement in renal outcomes (proteinuria or eGFR) was incompletely reported (14/15), but improvement was comparable in cases in which immunosuppression was used (5/7 vs. 7/7). Each of the two patients with AIN on renal biopsy who failed to improve with immunosuppression had crescentic pauci-immune GN.

When crescents were present on renal biopsy, immunosuppression was commonly prescribed (Appendix 2) (15/19, 78.9%). Renal outcomes (improvement in proteinuria or GFR) were incompletely reported but similarly improved in cases in which immunosuppression was used compared with those where immunosuppression was withheld (12/15 vs. 4/4). The three patients with crescents on renal biopsy who failed to improve with immunosuppression included two patients with anti-GBM disease and one patient with crescentic pauci-immune GN; all three of these patients remained persistently dialysis-dependent, despite immunosuppression with corticosteroids and plasmapheresis in all three cases, and additionally cyclophosphamide in the two cases of anti-GBM disease.

When granulomas were present on renal biopsy, immunosuppression was used in only one case (Appendix 2) (1/4, 25.0%). In this case of membranous nephropathy with AIN and granulomas [19], corticosteroids were added after initial improvement in proteinuria (from 7.6 g/day) was noted with anti-tuberculous therapy. Whether immunosuppression impacted renal outcomes in this case is thus uncertain.

### Specific types of glomerulonephritis

Cases of Glomerular disease in Tuberculosis were categorized by diagnosis based on renal pathology (Appendices 1 and 2).

#### Amyloidosis

This study is not designed to predict accurately the incidence of different types of glomerulonephritis in tuberculosis infection. Most of the cases (69/76) of amyloidosis in tuberculosis infection came from 2 cohort studies [20, 21]. Amyloidosis presented with AKI (54/74, 73.0%), nephrotic-range proteinuria (49/76, 64.5%), and hypertension (20/47, 42.6%) [15, 20–26]. Most cases of amyloidosis in this scoping review did not report whether AIN, crescents or granulomas were present; however, in those cases in which this information was reported, they were consistently absent (0/5). Amyloidosis was attributed to TB in most cases (75/76, 98.7%). Only one case used immunosuppression; in this case, a 25 year old man developed nephrotic syndrome, hypertension and AKI necessitating hemodialysis, simultaneous to the presentation of pulmonary TB. Initiation of monthly tocilizumab and anti-tuberculous therapy (ATT) led to improvement in renal function and dialysis independence. The subtype of amyloidosis (AA versus AL) was not consistently reported; tuberculosis infection was implicated in 90% of cases of AA amyloidosis in one case series from India [21], but in 51–65% of cases of other historic cohorts [27–29]. 

#### Anti-glomerular basement membrane disease glomerulonephritis

Cases of Anti-GBM GN presented with AKI (3/3, 100.0%) with 2 patients starting hemodialysis [30–32]. Nephrotic range proteinuria with clinical features of nephrotic syndrome was reported in 2 cases. Renal pathology revealed crescents (3/3), but granulomas and AIN were absent. All cases were attributed to TB. Immunosuppression was used in all cases, with cyclophosphamide + corticosteroids + plasmapheresis in all cases. Improvement in renal function was noted in one patient, but both patients who started hemodialysis remained dialysis dependent.

#### Crescentic (pauci-immune) glomerulonephritis

Cases of crescentic (pauci-immune) GN presented with AKI (10/10, 100.0%), hypertension (4/7 reported, 57.1%), and nephrotic syndrome (3/10, 30.0%) [4, 9, 32–39]. Most (7/10, 70.0%) cases were ANCA-negative, with the remainder Anti-Proteinase-3 (2/10) or Anti-Myeloperoxidase (1/10) positive. Two patients required initiation of dialysis at the time of diagnosis of crescentic GN; neither patient recovered renal function, despite cure of TB with ATT, and immunosuppression in both cases. Renal pathology revealed crescents (10/10, 100.0%), AIN (5/10, 50.0%) but no granulomas. Cases were most often attributed to TB (6/10, 60.0%), then to ATT (3/10, 30.0%). When attribution was to ATT, rifampin was the culprit medication in each case. Immunosuppression was used in most cases (8/10, 80.0%). In cases where immunosuppression was not used (2/10), one patient fully recovered renal function by stopping rifampin, while another patient fully recovered by curing TB with ATT. In total, full (4/10) or partial (2/10) recovery of renal function, persistent need for dialysis (2/10) and unknown long-term renal outcomes (2/10) were seen.

#### Focal segmental glomerulosclerosis

There were 2 cases of FSGS, both of which presented with nephrotic syndrome, hypertension, and AKI [11, 40]. Renal pathology did not reveal crescents, AIN or granulomas. Both cases were attributed to TB, but corticosteroids were prescribed in both cases. Improvement in renal function and proteinuria was noted in both cases, with timing coinciding better with corticosteroid therapy than with ATT in one case.

#### IgA nephropathy

IgA nephropathy presented with nephrotic syndrome (6/13, 46.2%) and AKI (6/13, 46.2%) [6, 12, 13, 34, 41–49]. Blood pressure was uncommonly reported (7/13), but hypertension was present in only a minority of reported cases (1/7, 14.3%). HSP-IgA nephritis was reported (3/13, 23.1%); in these cases, patients presented with AKI (2/3, 66.7%) and nephrotic syndrome (1/3, 33.3%). Renal pathology revealed AIN (1/13), crescents (2/13) and granulomas (1/13). Cases of HSP presented with AIN (1/3), crescents (2/3) and granulomas (1/3). IgA nephropathy was attributed to TB in most cases (11/13, 84.6%) although it often was unclear (2/13). Renal function and proteinuria improved in most cases (11/13) with ATT. In cases with corticosteroid use (3/13), initiation of corticosteroids coincided with improvement in renal outcomes in 2 cases.

#### Immunotactoid glomerulonephritis

In the only case of immunotactoid GN, a 37 year old man presented 9 weeks after TB diagnosis with nephrotic syndrome and anasarca, AKI and hypertension [50]. Renal pathology revealed no crescents, AIN or granulomas, and immunotactoid GN was attributed to TB. Proteinuria improved but did not completely resolve, with ATT (without immunosuppression).

#### Membranoproliferative glomerulonephritis

Cases of MPGN typically presented with AKI (5/5, 100.0%), hypertension (3/5, 60.0%) and nephrotic syndrome (3/5, 60.0%) [8, 51–54]. One case presented with anuric AKI, necessitating renal replacement therapy. Renal pathology revealed crescents in multiple (3/5, 60.0%) cases, but AIN and granulomas were not reported. MPGN was attributed to ATT in 1/5 cases which improved after holding rifampin and giving corticosteroids; however, MPGN was attributed to TB in all other cases (4/5, 80.0%). MPGN improved in all reported cases, 2 of which combined anti-tuberculous therapy with immunosuppression (Corticosteroids +/- mycophenolate mofetil).

#### Membranous nephropathy

Cases of membranous nephropathy presented with nephrotic syndrome (7/10, 70.0%), AKI (3/10, 30.0%) and hypertension (2/10, 20.0%) [7, 10, 14, 19, 55–60]. Renal pathology revealed AIN (6/10, 60.0%), and granulomas (3/10, 30.0%) but no crescents. Cases were attributed to TB (6/10), medication (2/10) or unclear (2/10). All cases with granulomas were attributed to TB. Anti-phospholipase-2-receptor (PLA2R) testing was reported in 2 cases, with one case being positive. In the case of anti-PLA2R positive membranous nephropathy, the GN was attributed to ATT, with full resolution of the nephrotic syndrome (5.3 g/day proteinuria) within 2 weeks of stopping Rifampin. Corticosteroids were used in three cases, and in 2 of these the GN was attributed to ATT. In all three cases, proteinuria improvement coincided with ATT, with uncertain impact of corticosteroid therapy.

#### Minimal change disease

Cases of minimal change disease typically presented with nephrotic syndrome (6/7, 85.7%), and AKI was common (4/7, 57.1%) [16, 61–66]. Hypertension was not reported in any case (0/7, 0.0%). Renal pathology revealed concurrent AIN in 3 cases (42.9%) but crescents and granulomas were not described. Minimal change disease was attributed to anti-tuberculous therapy in all cases, with rifampin or isoniazid identified as the culprit medication in all cases. Proteinuria improved in most cases by stopping the offending medication (6**/**7), and in two of three cases with corticosteroid use.

#### Mixed pathology

Three cases of mixed IgA/Membranous/MPGN pathology were identified, in which patients presented with AKI (3/3), hypertension (1/3), but without nephrotic syndrome (0/3) [67–69]. Renal pathology did not reveal AIN or granulomas. One case was attributed to rifampin, with resolution of GN within 1 month of stopping rifampin. In the other two cases, GN was attributed to TB, but renal outcomes with ATT were not noted in one case. Crescents were identified in one GN case attributed to TB; corticosteroids and cyclophosphamide were administered simultaneous with ATT with GN resolution later noted.

## Discussion

This review explores the presentation, diagnosis, management and outcomes of glomerular disease in *Mycobacterium tuberculosis* infections. The reported case reports and case series make up 62 studies, representing 130 patients. These cases included a spectrum of presentations that included AKI, nephrotic syndrome or hypertension, a range of 10 unique renal pathologies, cases that used immunosuppression, and a variety of outcomes ranging from complete remission to long-term dialysis dependence. This study thus represents the most comprehensive study of glomerular disease in TB infections.

When caring for a patient with TB and GN, a clinician must consider the utility of performing a renal biopsy. Unfortunately, patient presentation with nephrotic syndrome, hypertension or AKI was not specific to any of the 10 renal pathologies. However, there were specific features on renal pathology that were highly predictive of whether the GN was attributed to TB infection or anti-tuberculous therapy. When identified, granulomas (4/4), anti-GBM disease (3/3), amyloidosis (75/76), and FSGS (2/2) were highly specific for the etiology of GN being from TB infection. On the other hand, every case of minimal change disease (7/7) was attributed to ATT.

When ATT is implicated as the precipitant of GN, a change in therapy should be considered. In addition, depending on the renal pathology, such as patients with crescentic pauci-immune GN (*n* = 12), the addition of immunosuppression may also be considered. While this study is underpowered to establish the efficacy of immunosuppression in patients with TB, it is generally agreed that immunosuppression in such instances should be considered [70]. There are significant risks of immunosuppression, especially in patients with active TB infection; exposure of patients to this risk should only be considered if renal biopsy features suggest possible benefit.

When a patient has GN attributed to TB infection, clinicians may continue to use the most effective ATT, and to minimize patient exposure to second line therapies that may have increased toxicity, cost, and monitoring [71]. On the other hand, only isoniazid or rifampin were implicated when GN was determined to be secondary to anti-TB medications. Since both of these medications are considered the backbone of TB treatment and commonly prescribed as part of the standard first-line treatment regimen for TB, a high threshold must be met before modifying standard ATT.

Given the volume of cases identified in this study, coincidental primary GN cannot be ruled out, despite the timing of presentation suggesting a link with TB infection or ATT. The serum Anti-PLA2R antibody was checked in three cases of membranous nephropathy. In the only case in which Anti-PLA2R antibody was positive [60], the GN was attributed to the ATT, and indeed resolved upon ATT completion. Cases of Anti-PLA2R antibody positive secondary membranous nephropathy are well described [72], and production of pathogenic autoantibodies is increased by TB infection by persistent stimulation of the antibody-mediated immune response [73]. Therefore, the clinical utility of Anti-PLA2R antibodies to differentiate between primary and secondary infections may be reduced in patients with active TB infection.

TB modifies humoral mediated immunity by several mechanisms, predisposing to immune complex deposition and glomerulonephritis. Patients with TB may produce IgA antibodies against A-60 mycobacterial antigen, which activates lectin and alternative complement pathways [37]. Similarly, hypersensitivity reactions to TB wall antigens lead to immune complex deposition in the perivascular, peritubular, vascular and glomerular spaces. This ultimately leads to vasculitis, with the renal pathology determined in part by the mycobacterial cell wall antigen initiating the cascade [74]. Chronic infections including TB cause chronic stimulation of innate and adaptive immune responses through Interferon and Th1-immune response [73]. 

Rifampin and isoniazid-induced GN in patients with TB may arise from a variety of mechanisms. First, anti-rifampin antibodies have been described in patients with crescentic GN, and it is postulated that antibody specificity relates to renal pathology [75]. Intermittent gaps, then re-initiation in ATT may be followed by an intense immune reaction [76]. Secondly, medication may have direct toxin effects on glomeruli [77]. Thirdly, both cell-mediated and humoral immune responses secondary to rifampin have been described [16, 61]. While isoniazid has been described to precipitate a systemic necrotizing vasculitis [78], whether autoantibodies to isoniazid or a direct toxin effect on glomeruli precipitates GN remains uncertain [63]. 

This study has several strengths and weaknesses. To date, this is the most comprehensive review of GN cases in patients with TB. However, most studies were published in high income countries with correspondingly low TB rates. Therefore, cases from high TB prevalence regions, and cases of drug-resistant TB, are underrepresented. Attribution of GN to ATT versus TB is based on clinical judgment, and thus may be inaccurate in some cases. There was likely to have been reporting bias, with published cases more likely reflecting treatment successes than failures. Case reports and case series are subject to information bias including recall bias, and thus may be less representative and associated with limited or incomplete qualitative data. However, this study explored the link between renal pathology features (crescents, granulomas, AIN) and clinical presentation and outcomes. This study has reported renal pathologic features that may predict whether GN is related to anti-TB therapy versus TB infection, which has important treatment implications for clinicians. Important trends and predictive factors were identified despite the study limitations.

Patient demographics collected included patient age and sex. Clinical presentation data included timing of GN from TB symptoms and diagnosis, GN presentation symptoms, quantification of proteinuria, and presence of nephrotic syndrome, hypertension, or AKI.

Renal biopsy data included the pathologic diagnosis, whether the renal biopsy contained acute interstitial nephritis (AIN), granulomas or crescents, and whether the GN was attributed to TB or medications.

The attribution of GN to either TB or anti-TB medications was deferred to the included studies’ determination. When included studies did not attribute the GN to either TB or anti-TB medications, this was also recorded.

Management data collected included whether immunosuppression was prescribed (with regimen and duration if used), and modifications in anti-TB medications. Outcomes of the TB and renal disease were also collected.

### Ethics

This study is consistent with the principles outlined by the Committee on Publication Ethics. Health Sciences Research Ethics Board approval was not required, since all information used in this manuscript was already freely available in the public domain, and the dataset analyzed was properly anonymized with informed consent obtained at the time of original data collection.

### Registration

This trial was not registered since PROSPERO does not permit registration of scoping reviews.

### Electronic supplementary material

Below is the link to the electronic supplementary material.

Supplementary Material 1

## Data Availability

Database of studies is summarized in Appendices 1 and 2. However, raw data is available on request to the corresponding author.

## Abbreviations

AKI: Acute kidney injury
AIN: Acute interstitial nephritis
ANCA: Anti-neutrophil cytoplasmic antibody
ATT: Anti-tuberculous Therapy
eGFR: Estimated glomerular filtration rate
FSGS: Focal segmental glomerulosclerosis
GBM: Glomerular basement membrane
GN: Glomerulonephritis
HSP: Henoch-Schonlein purpura
HIV: Human Immunodeficiency Virus
LMIC: Low or Middle Income Country
MPGN: Membranoproliferative glomerulonephritis
PLA2R: Phospholipase-A2 receptor
TB: Tuberculosis
