# Supplementary material for: Glomerulonephritis during Mycobacterium tuberculosis infection: scoping review
Source: BMC Nephrol. 2024 Aug 31;25:285. doi: 10.1186/s12882-024-03716-6 (PMC11366146; doi:10.1186/s12882-024-03716-6)
Supplement: Supplementary file 1 — Supplementary Material 1 [file 12882_2024_3716_MOESM1_ESM.docx]

| **Appendix 1: Case Presentation** | | | | | | |  |  |
| --- | --- | --- | --- | --- | --- | --- | --- | --- |
| **Study Author** | **Number cases** | **Age (years)** | **Sex (0 = male; 1 = female)** | **Proteinuria (g/24 hours)** | **Nephrotic Syndrome (0=no, 1=yes)** | **Hypertension (0=no, 1=yes)** | **AKI (0=no, 1=yes)** | **TB Location (lung, cecum, etc)** |
| **AMYLOIDOSIS** | | | | | | | | |
| Akram 2020 | 1 | 32 | 0 | 3.7 | 1 | 0 | 1 | Lung |
| Dixit 2009 | 43 | Mean 38.4 | 32 male | ** | 23/43 | 19/43 | 32/43 | NS |
| El-Hennawy 2002 | 1 | 25 | 0 | 20.9 | 1 | NS | 0 | Cecum |
| Engineer, 2018 | 26 | NS | 20 male | *** | NS | NS | 20/26 | NS |
| Magro-Checa, 2011 | 1 | 25 | 0 | 18 | 1 | 1 | 1 | Lung |
| Mandal, 2013 | 1 | 22 | 0 | 4.5 | 1 | 0 | 0 | Lung |
| Monsel, 2011 | 1 | 33 | 0 | 4.2 | 1 | 0 | 0 | Anal |
| Rhodes, 1987- case 1 | 1 | 40 | 0 | 8.5 | 1 | NS | NS | NS |
| Rhodes, 1987- case 2 | 1 | 30 | 0 | 13.2 | 1 | NS | NS | NS |
|  |  |  |  |  |  |  |  |  |
| **ANTI-GLOMERULAR BASEMENT MEMBRANE DISEASE** | | | | | | | | |
| Hsieh, 2012* | 1 | 66 | 0 | 2.3 | 0 | NS | 1 | Lung |
| Kashif, 2013$ | 1 | 32 | 0 | 4.6 | 1 | 0 | 1 | Lung |
| Wen, 2013 | 1 | 72 | 0 | NS | 0 | NS | 1 | Lung |
|  |  |  |  |  |  |  |  |  |
| **CRESCENTIC (PAUCI-IMMUNE) GLOMERULONEPHRITIS** | | | | | | | | |
| Iijima, 2018~ | 1 | 50 | 1 | 2+ UA | 0 | 1 | 1 | Nodular erythema, lymphadenopathy |
| Kanodia, 2016 ! | 1 | 26 | 0 | NS | 0 | 0 | 1 | Lung |
| Murray, 1987! | 1 | NS | NS | >3 | 1 | NS | 1 | Lung |
| Muthukumar, 2002! | 1 | NS | NS | NS | 0 | NS | 1 | Lung |
| O'Brien, 2021@ | 1 | 42 | 1 | NS | 0 | NS | 1 | Lung |
| Oxley Oxland, 2018!* | 1 | 47 | 0 | 0.2 | 0 | NS | 1 | Lung |
| Rostami, 2021~ | 1 | 28 | 0 | 1 | 0 | 1 | 1 | Lung |
| Solak, 2013! | 1 | 31 | 1 | 9.8 | 1 | 0 | 1 | Lung |
| Wen, 2009! | 1 | 70 | 0 | NS | 0 | 0 | 1 | Lymphadenopathy, disseminated TB |
| Yoshioka, 2002! | 1 | 60 | 0 | 4.2 | 1 | 1 | 1 | Lung |
|  |  |  |  |  |  |  |  |  |
| **FOCAL SEGMENTAL GLOMERULOSCLEROSIS** | | | | | | | | |
| Rodriguez, 2010 | 1 | 61 | 0 | 9.2 | 1 | 1 | 1 | Lung |
| Srinivasaprasad, 2016 | 1 | 40 | 0 | 7.6 | 1 | 1 | 1 | Lung |
|  |  |  |  |  |  |  |  |  |
| **IgA NEPHROPATHY** | | | | | | | | |
| Audley 2022* | 1 | 41 | 0 | 0.4 | 0 | 0 | 1 | Lung, Spine |
| Bowman 1991 | 1 | 44 | 0 | 5.6 | 1 | NS | 0 | Lung |
| BuenoFilho 2012& | 1 | 45 | 1 | 0.3 | 0 | NS | 0 | Cutaneous, adenopathy |
| Cohen, 1985 | 1 | 59 | 0 | NS | 0 | 0 | 0 | Lung, Arthritis |
| De Siati, 1999 | 1 | 31 | 0 | 5.1 | 1 | NS | 1 | Lung |
| Han, 1995& | 1 | 41 | 0 | 6.9 | 1 | NS | 1 | Lung |
| Keven, 2004 | 1 | 36 | 0 | 6.8 | 1 | 0 | 0 | Lung |
| Kitamura, 2007& | 1 | 38 | 0 | 1.4 | 0 | NS | 1 | Lung |
| Matsuzawa, 2002 | 1 | 35 | 1 | 5.6 | 1 | 0 | 0 | Lung, abdominal |
| O'Brien, 1990 | 1 | 63 | 0 | 3.9 | 1 | 0 | 0 | Lung |
| Pradeep, 2019 | 1 | 72 | 0 | 1+ UA | 0 | NS | 1 | Renal |
| Shribman, 1983 | 1 | 45 | 0 | 1.3 | 0 | 1 | 1 | Lung, Liver |
| Singh, 2009 | 1 | 36 | 0 | 2.2 | 0 | 0 | 0 | Lung/Pleural |
|  |  |  |  |  |  |  |  |  |
| **IMMUNOTACTOID GLOMERULOPATHY** | | | | | | | | |
| Gupta, 2011 | 1 | 37 | 0 | 4.8 | 1 | 1 | 0 | Lung |
|  |  |  |  |  |  |  |  |  |
| **MEMBRANOPROLIFERATIVE DISEASE** | | | | | | | | |
| Helvaci, 2021 | 1 | 53 | 0 | 3.7 | 1 | 1 | 1 | Lung, lymphadenopathy |
| Kistler, 1999 | 1 | 69 | 1 | 13.4 | 1 | NS | 1 | Lung |
| Meyrier, 1988 | 1 | 46 | 0 | 8 | 1 | 1 | 1 | Lung, liver |
| Ram, 2014 | 1 | 48 | 0 | 1.4 | 0 | 1 | 1 | Spine |
| Samuel, 2017 | 1 | 82 | 1 | NS | 0 | NS | 1 | Lung |
|  |  |  |  |  |  |  |  |  |
| **MEMBRANOUS NEPHROPATHY** | | | | | | | | |
| Balwani 2021^ | 1 | 30 | 1 | 5.3 | 1 | NS | 0 | Lung |
| Gabow, 1976 | 1 | 49 | 0 | 2+ UA | 0 | NS | 1 | Lung |
| Ghosh, 2011 | 1 | 24 | 1 | 1.8 | 0 | 0 | 0 | Peritoneal |
| Malhotra, 2019 | 1 | 32 | 0 | 9 | 1 | 0 | 0 | Renal |
| Morimoto, 2022# | 1 | 50 | 1 | 6 | 1 | 1 | 0 | Lung |
| Pal, 2022 | 1 | 42 | 0 | 7.6 | 1 | 0 | 0 | Renal |
| Ram, 2011 | 1 | 23 | 1 | >3 | 1 | NS | 0 | NS |
| Rodriguez-Garcia, 1990# | 1 | 66 | 0 | 3 | 1 | NS | 0 | Lung |
| Suzuki, 1989 | 1 | 63 | 1 | 4.5 | 1 | 0 | 1 | Renal |
| Yuan, 2010 | 1 | 50 | 1 | 2.6 | 0 | 1 | 1 | Spinal |
|  |  |  |  |  |  |  |  |  |
| **MINIMAL CHANGE DISEASE** | | | | | | | | |
| Barnes 1984 | 1 | 52 | 0 | 1.9 | 0 | 0 | 1 | Lung |
| Kim, 2018 | 1 | 51 | 1 | 12.2 | 1 | 0 | 0 | Lung |
| Mori, 2011 | 1 | 66 | 1 | 12 | 1 | NS | 1 | Lung (latent) |
| Neugarten, 1983 | 1 | 72 | 0 | 4.1 | 1 | 0 | 1 | Lung |
| Park, 2014 | 1 | 68 | 1 | 9.2 | 1 | NS | 1 | Lung/Pleural |
| Sathi, 2021 | 1 | 26 | 0 | 10.8 | 1 | NS | 0 | Lung |
| Tada, 1995 | 1 | 53 | 0 | 4.8 | 1 | 0 | 0 | Lung |
|  |  |  |  |  |  |  |  |  |
| **MIXED PATHOLOGY** | | | | | | | | |
| Isotalo, 2002 | 1 | 28 | 1 | NS | 0 | NS | 1 | Lung |
| Sopena 1991 | 1 | 55 | 0 | 0.7 | 0 | 1 | 1 | Lung |
| Yong, 2002 | 1 | 28 | 1 | NS | 0 | NS | 1 | Lung |
| * proteinuria was reported in Urine Protein to creatinine ratio, and converted to 24 hour urine, using Mayayo MM et al, 2016  ** (Dixit, 2009): 23 patients had nephrotic range proteinuria, and 23 also had nephrotic syndrome. 15 of these patients had > 10 g/day proteinuria  *** (Engineer, 2018): AA Amyloidosis in 26 patients with TB. 19 of these 26 had nephrotic range proteinuria. The AA amyloidoisis from TB were a subset of 40 patients with AA, AL and undetermined amyloidosis, 3 of whom had nephrotic range proteinuria without nephrotic syndrome (not reported if AA/AL/undetermined). Thus, presence of nephrotic syndrome couldn’t be established. | | | | | | | | |
| $=This case was Anti-GBM and ANCA positive; &=These cases were IgA Nephropathy with Henoch-Schonlein Purpura | | | | | | | | |
| ^=Anti-PLA2R antibody positive; #=Anti-PLA2R negative  ! = ANCA-negative; ~ = Anti-PR3 positive; @ = Anti-MPO positive | | | | | | | | |
| **LEGEND:** NS = not stated; UA = urinalysis | | | | | | | | |

| **Appendix 2: Renal Pathology Features, Management and Outcomes** | | | | | | | | | |
| --- | --- | --- | --- | --- | --- | --- | --- | --- | --- |
|  |  |  | **Renal Biopsy Features 0 = no, 1 = yes** | | |  |  |  |  |
| **Study Author** | **Number cases** | **Cause of GN Medication = 0 TB=1 Uncertain = 2** | **AIN** | **Crescents** | **Granuloma** | **Immunosuppression** | **Anti-TB therapy** | **Change in GN 0=no change, 1=improve, 2=worsening** | **Relevant Additional Details** |
| **AMYLOIDOSIS** | | | | | | | | | |
| Akram 2020 | 1 | 1 | 0 | 0 | 0 | None | RIPE | 2 | Patient died 1 month from presentation |
| Dixit 2009 | 43 | 1 | NS | NS | NS | NS | NS | NS | None provided |
| El-Hennawy 2002 | 1 | 1 | 0 | 0 | 0 | None | NS | NS | None provided |
| Engineer, 2018 | 26 | 1 | NS | NS | NS | None | NS | NS | None provided |
| Magro-Checa, 2011 | 1 | 1 | 0 | 0 | 0 | TOC | I | 1 | GN improved with TOC initiation |
| Mandal, 2013 | 1 | 0 | 0 | 0 | 0 | None | NS | 1 | None provided |
| Monsel, 2011 | 1 | 1 | 0 | 0 | 0 | None | RIPE | 1 | Full resolution to normal renal function |
| Rhodes, 1987 | 2 | 1 | NS | NS | NS | None | NS | NS | None provided |
| Rhodes, 1987 | 2 | 1 | NS | NS | NS | None | NS | NS | None provided |
|  |  |  |  |  |  |  |  |  |  |
| **ANTI-GLOMERULAR BASEMENT MEMBRANE DISEASE** | | | | | | | | | |
| Hsieh, 2012* | 1 | 1 | 0 | 1 | 0 | CORT, CYC, PLEX | RIPE | 0 | Remained dialysis dependent |
| Kashif, 2013$ | 1 | 1 | 0 | 1 | 0 | CORT, CYC, PLEX | RIPE | 1 | 6 months follow-up, Serum Cr 1.2 mg/dL |
| Wen, 2013 | 1 | 1 | 0 | 1 | 0 | CORT, CYC, PLEX | NS | 0 | Remained dialysis dependent |
|  |  |  |  |  |  |  |  |  |  |
| **CRESCENTIC (PAUCI-IMMUNE) GLOMERULONEPHRITIS** | | | | | | | | | |
| Iijima, 2018~ | 1 | 2 | 1 | 1 | 0 | CORT | RIPE | 0 | CKD after ATT completed. |
| Kanodia, 2016! | 1 | 1 | 1 | 1 | 0 | CORT, IVIG, PLEX | RIPE | 2 | Remained dialysis dependent |
| Murray, 1987! | 1 | 0 | 1 | 1 | 0 | None | RIPS | 1 | None provided |
| Muthukumar, 2002! | 1 | 0 | 0 | 1 | 0 | CORT | R+? | 1 | None provided |
| O'Brien, 2021@ | 1 | 1 | 1 | 1 | 0 | CORT, MMF | RIPE | 1 | Full resolution to normal renal function |
| Oxley Oxland, 2018!* | 1 | 1 | 0 | 1 | 0 | CORT, CYC | RIPE | 1 | Full resolution to normal renal function |
| Rostami, 2021~ | 1 | 1 | 0 | 1 | 0 | CORT, CYC, then AZA | RIPL | 2 | Remained dialysis dependent |
| Solak, 2013! | 1 | 1 | 0 | 1 | 0 | CORT | RIPE | 1 | Proteinuria resolved, renal function improved |
| Wen, 2009! | 1 | 1 | 0 | 1 | 0 | None | RIPE | 1 | Full resolution to normal renal function |
| Yoshioka, 2002! | 1 | 0 | 1 | 1 | 0 | CORT | RIPE | 1 | Full resolution stopping RIF+starting CORT |
|  |  |  |  |  |  |  |  |  |  |
| **FOCAL SEGMENTAL GLOMERULOSCLEROSIS** | | | | | | | | | |
| Rodriguez, 2010! | 1 | 1 | 0 | 0 | 0 | CORT | RIP | 1 | Improvement with CORT not ATT |
| Srinivasaprasad, 2016! | 1 | 1 | 0 | 0 | 0 | CORT | RIPE | 1 | Recovered dialysis independence |
|  |  |  |  |  |  |  |  |  |  |
| **IgA NEPHROPATHY** | | | | | | | | | |
| Audley 2022* | 1 | 1 | 0 | 1 | 0 | None | RIPE | 1 | None provided |
| Bowman 1991 | 1 | 2 | 0 | 0 | 0 | CORT | RIPE | 0 | Persistent nephrotic range albuminuria |
| BuenoFilho 2012& | 1 | 1 | 0 | 0 | 0 | None | RIPE | 0 | Persistent hematuria and proteinuria |
| Cohen, 1985 | 1 | 1 | 0 | 0 | 0 | None | RI | 1 | GN resolved as did normal renal function |
| De Siati, 1999 | 1 | 1 | 0 | 0 | 0 | None | RI | 1 | Full resolution to normal renal function |
| Han, 1995& | 1 | 1 | 1 | 1 | 1 | None | RIPE | 1 | Full resolution to normal renal function |
| Keven, 2004 | 1 | 1 | 0 | 0 | 0 | None | RIPE | 1 | Full resolution to normal renal function |
| Kitamura, 2007& | 1 | 2 | 0 | 1 | 0 | CORT | RIPE | 1 | GN improved with CORT initiation |
| Matsuzawa, 2002 | 1 | 1 | 0 | 0 | 0 | None | NS | 1 | None provided |
| O'Brien, 1990 | 1 | 1 | 0 | 0 | 0 | None | NS | 1 | Full resolution to normal renal function |
| Pradeep, 2019 | 1 | 1 | 0 | 0 | 0 | None | RIPL | 1 | Full resolution to normal renal function (required ureteric stenting for genitourinary TB also) |
| Shribman, 1983 | 1 | 1 | 0 | 0 | 0 | CORT | NS | 1 | Full resolution of proteinuria but CKD |
| Singh, 2009 | 1 | 1 | 0 | 0 | 0 | None | RIPE | 1 | Resolution of proteinuria |
|  |  |  |  |  |  |  |  |  |  |
| **IMMUNOTACTOID GLOMERULOPATHY** | | | | | | | | | |
| Gupta, 2011 | 1 | 1 | 0 | 0 | 0 | None | RIPE | 1 | Improvement without full resolution |
|  |  |  |  |  |  |  |  |  |  |
| **MEMBRANOPROLIFERATIVE GLOMERULONEPHRITIS** | | | | | | | | | |
| Helvaci, 2021 | 1 | 1 | 0 | 1 | 0 | CORT, MMF | RIPE | 1 | Full resolution to normal renal function |
| Kistler, 1999 | 1 | 0 | 0 | 1 | 0 | CORT | RIP, then IPC | 1 | GN improved after RIF stopped |
| Meyrier, 1988 | 1 | 1 | 0 | 0 | 0 | None | RIE | 1 | Full resolution to normal renal function |
| Ram, 2014 | 1 | 1 | 0 | 0 | 0 | None | RIPL | 1 | Proteinuria improved with ATT |
| Samuel, 2017 | 1 | 1 | 0 | 1 | 0 | None | RIPE | NS | TB developed in context of withdrawal of Adalumumab for Rheumatoid arthritis |
|  |  |  |  |  |  |  |  |  |  |
| **MEMBRANOUS NEPHROPATHY** | | | | | | | | | |
| Balwani 2021^ | 1 | 0 | 0 | 0 | 0 | None | RIPE | 1 | GN improved after ATT completed |
| Gabow, 1976 | 1 | 0 | 1 | 0 | 0 | None | RIPE | 1 | GN resolved after RIF stopped |
| Ghosh, 2011 | 1 | 1 | 0 | 0 | 0 | None | RIPE | 1 | None provided |
| Malhotra, 2019 | 1 | 1 | 1 | 0 | 0 | None | NS | 1 | Full resolution of hematuria and proteinuria with ATT |
| Morimoto, 2022# | 1 | 2 | 0 | 0 | 0 | None | NS | 1 | Patient had simultaneous diagnosis of TB and Lung adenocarcinoma |
| Pal, 2022 | 1 | 1 | 1 | 0 | 1 | CORT | RIPE | 1 | Proteinuria improving 1 month follow-up |
| Ram, 2011 | 1 | 1 | 1 | 0 | 1 | None | NS | 1 | Full resolution to normal renal function |
| Rodriguez-Garcia, 1990# | 1 | 1 | 0 | 0 | 0 | CORT | RI | 1 | Improved with ATT |
| Suzuki, 1989 | 1 | 1 | 1 | 0 | 1 | None | RI | 1 | Resolution of proteinuria |
| Yuan, 2010 | 1 | 2 | 1 | 0 | 0 | CORT | RIPE | 1 | Improvement of GN with CORT. ATT timing unclear |
|  |  |  |  |  |  |  |  |  |  |
| **MNIMAL CHANGE DISEASE** | | | | | | | | | |
| Barnes 1984 | 1 | 0 | 1 | 0 | 0 | None | RIPE | 1 | Renal function normalized |
| Kim, 2018 | 1 | 0 | 0 | 0 | 0 | CORT | RI | NS | None provided |
| Mori, 2011 | 1 | 0 | 0 | 0 | 0 | CORT | I | 1 | GN resolved with CORT |
| Neugarten, 1983 | 1 | 0 | 1 | 0 | 0 | None | RIP | 1 | Resolved with stopping RIF |
| Park, 2014 | 1 | 0 | 0 | 0 | 0 | None | RIPE | 1 | Full resolution to normal renal function, with stopping RIF |
| Sathi, 2021 | 1 | 0 | 0 | 0 | 0 | None | RIPE, then IPEL | 1 | Resolution of proteinuria |
| Tada, 1995 | 1 | 0 | 1 | 0 | 0 | CORT | RIPE | 1 | Improvement with holding RIF, then (2 weeks later) starting CORT |
|  |  |  |  |  |  |  |  |  |  |
| **MIXED PATHOLOGY** | | | | | | | | | |
| Isotalo, 2002 | 1 | 1 | 0 | 0 | 0 | None | RIPE | NS | None provided |
| Sopena 1991 | 1 | 1 | 0 | 1 | 0 | CORT, CYC | RIE | 1 | Incomplete resolution to CKD |
| Yong, 2002 | 1 | 0 | 0 | 0 | 0 | None | RIPE | 1 | Full resolution 1 month stopping RIF |
| *=proteinuria was reported in Urine Protein to creatinine ratio, and converted to 24 hour urine, using Mayayo MM et al, 2016 | | | | | | | | | |
| $=This case was Anti-GBM and ANCA positive; &=These cases were IgA Nephropathy with Henoch-Schonlein Purpura | | | | | | | | | |
| ^=Anti-PLA2R antibody positive; #=Anti-PLA2R antibody negative  ! = ANCA-negative; ~ = Anti-PR3 positive; @ = Anti-MPO positive | | | | | | | | | |
| **LEGEND:** | | | | | | | | | |
| AIN = acute interstitial nephritis; ATT = anti-tuberculosis treatment; AZA = azathioprine; CORT = corticosteroids; CYC = cyclophosphamide; GN = glomerulonephritis; | | | | | | | | | |
| IVIG = intravenous immunoglobulin; MMF = mycophenolate mofetil; NA = not applicable; NS = not stated; PLEX = plasmapharesis; RIF = rifampin; | | | | | | | | | |
| TB = tuberculosis; TOC = tociluzumab; UA = urinalysis | | | | | | | | | |
| **Anti-TB therapy**: C = clarithromycin; E = ethambutol; I = isoniazid; L = levofloxacin; P = pyrazinamide; R = rifampin; S = streptomycin | | | | | | | | | |
